# Isolation, modification and characterisation of cellulose from wild *Dioscorea bulbifera*

**DOI:** 10.1038/s41598-020-78533-6

**Published:** 2021-01-13

**Authors:** Joseph Kolawole Ogunjobi, Olayinka Mary Balogun

**Affiliations:** Department of Chemistry, The Federal University of Technology, PMB 704, Akure, Nigeria

**Keywords:** Biosynthesis, Environmental chemistry, Green chemistry, Organic chemistry, Polymer chemistry

## Abstract

This study attempted to valorise wild *Dioscorea bulbifera* yam presently known to be poisonous and inedible obtained from three sources. The functional properties as well as its chemical composition were determined and isolated. Isolated cellulose was modified by the actions of sodium hydroxide and maleic anhydride. The biomass, isolated products and modified cellulose were characterized. Results showed that the highest cellulose obtained was 82.6%. FT-IR results showed successful modification of cellulose with the presence of a carbonyl (C = O) adsorption band at around 1725 cm^-1^. SEM images and XRD data showed a clear decrease in crystallinity but a slight increase in crystallite size after modification of the mercerized cellulose. TGA results showed the polymers degraded between 284 and 414 °C. The study reveals that the modified cellulose has potential application as an adsorbent and industrial material.

## Introduction

*Dioscorea bulbifera* is one of the species of yam that is widely distributed in Africa and Asia. Commonly called aerial yam, it is cultivated and serves as yam food in West Africa, West Indies, South East Asia, South Pacific and the Carribean Islands. Apart from being consumed as food, *D. bulbifera* is known for its antioxidant and antifungal activities^1,2^. The wild uncultivated varieties of *D. bulbifera* occur as aggressive weeds in virgin lands and forests. Their bulbils are brownish in colour having irregular shape and dotted/rough surface. Unlike the cultivated ones that are edible, the wild varieties are known to be poisonous containing dioscorine and dihydrodioscorine^3^. Hence the bulbils are found littering farmland and wasting away in virgin lands.

In the context of the principles of green chemistry, utilisation of agricultural wastes for production of bio-based chemicals has been on the increase in the past two decades. From corn stalk, rice straw, oilseed rape, plantain peels to bagasse among many others, various chemicals and materials having green credentials have been prepared^4–7^. The wild *D. bulbifera* being a non-edible renewable resource and a highly invasive plant, could serve as a cheap source of cellulose and cellulose derivatives. Cellulose is the most highly wanted of all the chemicals derivable from agricultural wastes because of its abundance and the potentials to meet high demands. Although the natural/unmodified cellulose has found applications as sorbents for heavy metal ions and dyes uptake, chemical modification of the cellulose has been reported to have imparted improved adsorption capacity, physical and chemical properties affording spectrum of applications including metals recovery from aqueous solutions^8–10^. Modification of cellulose using succinic anhydride and maleic anhydride as well as mercerization to open up cellulose microfibrils are well documented^8,10,11^. Reaction of these reagents with cellulose provides a cheap and easy way of introducing carboxylic group functionality on the cellulose, which can subsequently be applied to capture heavy metal ions and dyes from aqueous solutions.

Most of the recently reported studies on the edible *D. bulbifera* centred on its nutritional properties, phytochemical composition, toxicity and bitterness^12–16^, physicochemical properties of its composite with cassava flour^17^, and elemental composition of the tuber^18^. No literature yet exists on biochemical composition and a possible attempt to valorise the wild variety of *D. bulbifera*. Therefore, this study looks into the functional properties (moisture content, pH, bulk and tapped density) of wild *D. bulbifera*, determines its lignocellulosic components (extractives, cellulose, hemicellulose and lignin); modifies and characterises isolated cellulose derived from the biomass.

## Materials and methods

### Sample collection

*D. bulbifera* was collected at three different locations: Ekiti, Isarun and Ondo road in Nigeria. The samples were first washed with clean water, sun dried and subsequently separated into bulbs and peels. The bulbs were thinly sliced and both samples sun dried for two weeks and dried again in an oven at 70 °C for 4 h. The two samples (bulbs and peels) were separately milled with a BLG 450 blender, sieved through 2 mm BS mesh and stored in air tight containers for analysis.

### Methods

#### Determination of extractive matter from biomass

Air dried ground sample (2 g) was weighed and wrapped in a filter paper and placed in a thimble of 250 ml soxhlet extractor. The sample was pre-treated separately with different solvents (acetone, ethanol–benzene and ethanol-cyclohexane mixture of 2:1). The solvent (60 ml:120 ml) was added into 250 ml flat bottom flask and heated to reflux at 100 °C for 7 h in accordance with ^19^. The resulting mixture was cooled for 10 min and the solvent recovered by distillation. The residue from the distillation called extractive was poured into a 250 ml beaker of known weight and dried in an oven at 105 °C. The extractive free sample was removed from the thimble, dried for 10 min at 105 °C in an oven and air dried until it was free of solvent. The extractive was calculated accordingly.

#### Determination of lignin content

Klason lignin is defined as the component insoluble in a 72% sulphuric acid solution. Ethanol–benzene extracted sample (1 g) was placed in a 100 ml beaker and treated with 15 ml 72% sulphuric acid solution while stirring and macerating the sample with a glass rod for 2 h at room temperature. The sample was diluted with water to a total volume of 675 ml and further boiled for 4 h. The resulting solution was allowed to settle, filtered and the filtrate was kept for the determination of acid-soluble lignin. The residue (acid-insoluble fraction) obtained was washed with copious amount of hot water until reaching a neutral pH and dried to a constant weight at 105 °C in an oven. The acid insoluble lignin was calculated accordingly following standard ^20^.

#### Determination of holocellulose content

Oven dried extractive- free biomass (2 g) was weighed, placed in a 250 ml beaker and treated by heating with 160 ml 1 M sodium acetate solution at 75 °C for 5 h. 0.11 M Sodium chlorite (4 ml) was added every hour during 4 h. The mixture was thereafter cooled, filtered and the residue washed with 1000 ml of distilled water and with 15 ml of acetone. The residue was finally dried at room temperature while an aliquot was weighed and dried at 105 °C for the determination of the holocellulose content.

#### Determination of α-cellulose content

About 2 g of holocellulose insoluble residue was transferred into a 250 ml beaker, 100 ml of 17.5 wt% sodium hydroxide solution was added at room temperature for 30 min incubating period. The residue was filtered and washed firstly two times with 200 ml of water and then filtered again. Then, 15 ml 10% acetic acid solution was added to the residue, afterward the residue was filtered and washed with 500 ml of hot water and dried at 105 °C in an oven. α-cellulose was finally determined gravimetrically.

#### Determination of pH in biomass

Approximately 0.1 g of the sample was boiled in a 250 ml beaker containing 100 ml hot distilled water for 5 min. The solution was diluted to 200 ml with distilled water and cooled at room temperature. Thereafter, pH of the biomass was taken using a pH meter (pH-107).

#### Determination of bulk and tapped density

Bulk density (B_d_) was determined as untapped free settled density. 1 g of the biomass was weighed into a 10 ml graduated cylinder and the volume occupied read. Tapped density (T_d_) was determined by tapping the base of the cylinder 50 times with a finger to compact the biomass until the weight of the sample was at a minimum and the volume read. The densities were determined as the ratio of the weight to volume of sample.

#### Determination of moisture content in biomass

An empty evaporating dish was washed and dried in the oven at 105 °C, cooled in a desiccator and weighed. 1 g sample of the biomass was added to the pre-weighed evaporating dish, dried for 3 h in the oven (DHG-OA Jino-tech instrument) at 105 °C. The resulting biomass was cooled in a desiccator and weighed. The evaporating dish and its content were returned to the oven for 1 h; repeating the cooling and weighing as above for successive hourly periods until constant weight was reached.

#### Cellulose mercerization

Cellulose (3.5 g) was treated with 200 ml NaOH solution (20 wt. %) at room temperature for 16 h with magnetic stirring at 5 rpm. The alkali-cellulose was separated from the solution using centrifuge (centrifuge 80-3, PEC Medical USA) and washed with distilled water down to pH 7 and then acetone. The mercerized product was dried at 90 °C in an oven for 1 h and stored in a desiccator ^11^.

#### Esterification of cellulose

Modification process for cellulose (treated or mercerized) was conducted in a round bottomed flask of 50 ml corked with a two-neck adapter on a magnetic stirrer equipped with a reflux condenser and a thermometer at constant temperature of 60 °C. Cellulose material (0.78 g) was introduced separately into the flask containing 50 ml of maleic anhydride solution in acetone (0.1 mold m^-3^). The esterification process was conducted for 8 h. Once the esterification was completed the product was filtered, washed several times with 330 ml of distilled water and thereafter extracted in a Soxhlet apparatus for 8 h using acetone. The modified cellulose was dried at room temperature for 48 h^21^.

#### Characterization of feedstocks and products

### Fourier transform infrared (FTIR) spectroscopic analysis

FTIR of the samples were prepared by mixing 1 mg of each material with 100 mg of spectroscopy grade KBr. The FTIR spectra were recorded at 4 cm^-1^ resolution from 4,400 to 350 cm^-1^ and 32 scans per sample using BX Perkin Elmer FT-IR spectrometer.

### X-ray diffractometry analysis

The crystalline structure of cellulose and modified cellulose samples were analysed by wide angle x-ray diffraction using XRD-6000 instruments. The diffractogram of isolated cellulose and modified cellulose from *D. bulbifera* were collected in the scattering angle 2Ɵ from 4° to 75° at the rate of 0.034°/s. The crystallinity index (CI) of the celluloses was determined with Origin 2019b software package using the following equation

$${\text{CI}}(\% ) = \frac{{{\text{A}}_{{{\text{crystalline}}}} }}{{{\text{A}}_{{{\text{crystalline}}}} + {\text{A}}_{{{\text{amorphous}}}} }} \times 100$$

where A_amorphous_ is the area under the amorphous curve, and A_crystalline_ is the area under the sample curve.

The cellulose crystallite size was estimated using Scherrer’s equation

$${\text{Crystal size L }} = k\lambda /\beta {\text{ cos}}\theta$$

where λ = 0.1540 nm, k is the correction factor of 0.91, θ is the diffraction angle in radians and β is the full width at half maximum.

### Scanning electron microscopy (SEM)

Surface morphology and inorganic composition of the samples were recorded using SEM JSM-6390LV coupled with energy-dispersive X-ray (EDX) 6733B-1UUS-SN equipment.

### Thermo-gravimetric analysis (TGA)

TGA of the samples were carried out using Perkin Elmer instrument. For each measurement, approximately 25 mg of the sample was used. Patterns were recorded under a nitrogen atmosphere at a flow rate of 100 mL/min by heating the material from room temperature to 900 °C at a heating rate of 20 °C/min.

### Statistical analysis

Data obtained were subjected to analysis of variance (ANOVA) using SPSS version 16.0 and the means were compared by Duncan’s New Multiple Range Test at 95% confidence level and 5% significance level.

## Results and discussion

### Chemical composition of lignocellulose biomass

**Figure 1:**
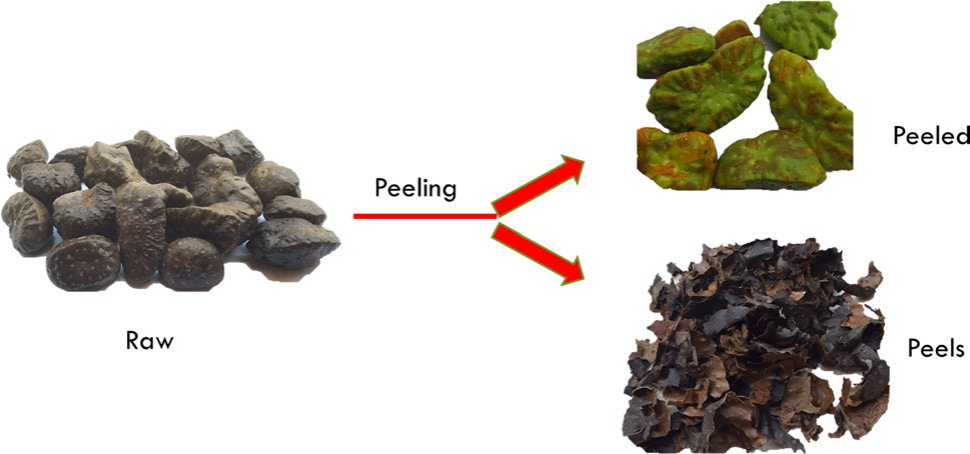
Raw wild *D. bulbifera* carefully separated into peels and peeled feedstock.

The peels and bulbils of samples from Isarun and Ekiti (IP, IB, EP and EB respectively) were extracted separately while it was difficult to peel the samples from Ondo thus both the peels and bulbils were extracted together as OPB. Typically, they are characterized by holocellulose content, cellulose content, hemicellulose content, lignin content and low extractives. The raw wild *D. bulbifera* when freshly peeled was green but turned brown afterwards when exposed to the atmosphere as shown in Fig. 1.

Percentage compositions of the lignocellulosic biomass of *D. bulbifera* collected from different locations are presented in Table 1 and discussed hereafter.

**Table 1:** Chemical Composition of wild *D. bulbifera* Biomass (wt% on dry matter).

Biomass	Holocellulose	Cellulose	Hemicellulose	Klason lignin	Extractive
EP	83.76^d^ ± 0.21	46.08^b^ ± 0.13	37.68^d^ ± 0.17	20.27^b^ ± 0.64	6.87^d^ ± 0.15
EB	80.43^c^ ± 0.59	30.74^a^ ± 0.26	49.66^e^ ± 0.37	49.90^c^ ± 0.10	3.63^b^ ± 0.15
IP	89.45^e^ ± 0.44	82.60^e^ ± 0.44	6.85^a^ ± 0.48	23.23^b^ ± 2.81	5.50^c^ ± 0.10
IB	69.00^b^ ± 0.56	49.80^c^ ± 0.10	18.93^c^ ± 0.95	9.70^a^ ± 0.26	1.84^a^ ± 0.04
OPB	62.43^a^ ± 0.45	52.70^d^ ± 0.26	9.73^b^ ± 0.29	12.00^a^ ± 2.65	13.90^e^ ± 0.10

Means ± standard deviation of triplicate determinations. Means within a column with the same superscript letters are not significantly different (p > 0.05) by Duncan’s multiple range test.

*EP* Ekiti peels of *Dioscorea bulbifera,* *EB *Ekiti bulbils of *Dioscorea bulbifera,* *IP* Isarun peels of *Dioscorea bulbifera,* *IB* Isarun bulbils of *Dioscorea bulbifera, OPB* Ondo peels and bulbils of *Dioscorea bulbifera.*

### Cellulose content of lignocellulose biomass

The analysis in Table 1 revealed the percentage composition of the cellulose content in the biomass. IP recorded the highest cellulose content value of 82.6% followed by OPB (52.7%), IB (49.8%), EP (46.08%) and then EB (30.74%). According to a rating system which stated that “any plant materials with equal or higher than 34% cellulose content are classified to be pulp and paper industrial raw material”^22^, it implies that all the samples except EB showed potentials as raw material suitable for pulp and paper manufacturing. Even with an upward review of the rating which placed a benchmark of 40% of cellulose content^23^, all the samples still meet the expectation and satisfactory cellulose content for pulp production. The cellulose contents obtained in samples studied are higher than or compared with those reported for other agricultural wastes elsewhere: almond shell, 21.7%; corn stalk, 35%; oil palm bark and shell, 18.85–29.7%; and bagasse, 40%^24–27^.

As revealed from statistical analysis result in Table 1, the cellulose contents of the biomasses are significantly different from each other. The chemical composition of *D. bulbifera* varies from sample to sample which could be due to difference in, species, geographical conditions, types of paddy, age and chemical used and these variations influence each polymer component of lignin, cellulose and hemicellulose as observed in the yield of cellulose obtained in this study.

#### Lignin content of lignocellulosic biomass

Variation in lignin composition is a function of plant species, age and tissue type. In Table 1, it was observed that Klason lignin was 9.7% in IB, 12.0% in OPB, 20.26% in EP, 23.2% in IP and 49.9% in EB. The results obtained in this study are close to the values reported previously for lignin content from other types of biomass wastes: Siam weed, rice straw, bagasse, sponge gourd, plantain peels and cocoa pods ^6,26,28,29^. The lignin content of the samples under observation, with the exception of EB, are < 30% and are within the range of the expected and satisfactory level for pulp production^30^. Conversely, lignin content > 20% is a good source of lignin that could be utilized for industrial purposes^31^. Therefore, the lignin content reported for EB is significantly high and could be converted to an economic value.

#### Holocellulose and hemicellulose contents of the biomass

Table 1 shows the results of holocellulose content ranging between 62.43% and 89.45%. Specifically, IP had the highest (89.45%) followed by EP (83.76%), EB (80.43%), IB (69.00%) and the least recorded in OPB (62.43%). The results were close to those reported elsewhere^32^. High holocellulose content correlates to a higher pulp yield which is desirable for pulp and paper production.

It was observed that the hemicellulose content obtained by subtracting the corrected cellulose from the corrected holocellulose content of the sample material ranged from 6.85 to 49.66% as presented in Table 1. High hemicellulose content could be taken as additive in cellulosic pulp which can help improve some mechanical properties of the paper, among other features of paper making in the paper industries^33^.

#### Extractive content of lignocellulosic biomass

The results of the ethanol–benzene solubility ranged from 13.90 to 1.84%. Table 1 shows that OPB has the highest extractive (13.90%) due to the fact that both the peels and bulbils are combined in the sample. EP has 6.87%, IP 5.50%, EB 3.63% and IB recorded the lowest value (1.84%). The high extractive content recorded for OPB in this study agrees with those reported for non-woody plants while the results for EB, IP, EB and IB agreed with values reported for woody plants ^34,35^. Comparatively, *D*. *bulbifera* from Ekiti had more extractive than those sourced from Isarun. In addition, results showed that the extractives are more concentrated in the peels than in the bulbils for both samples from Ekiti and Isarun.

#### Determination of extractive content using Greener solvents

**Figure 2:**
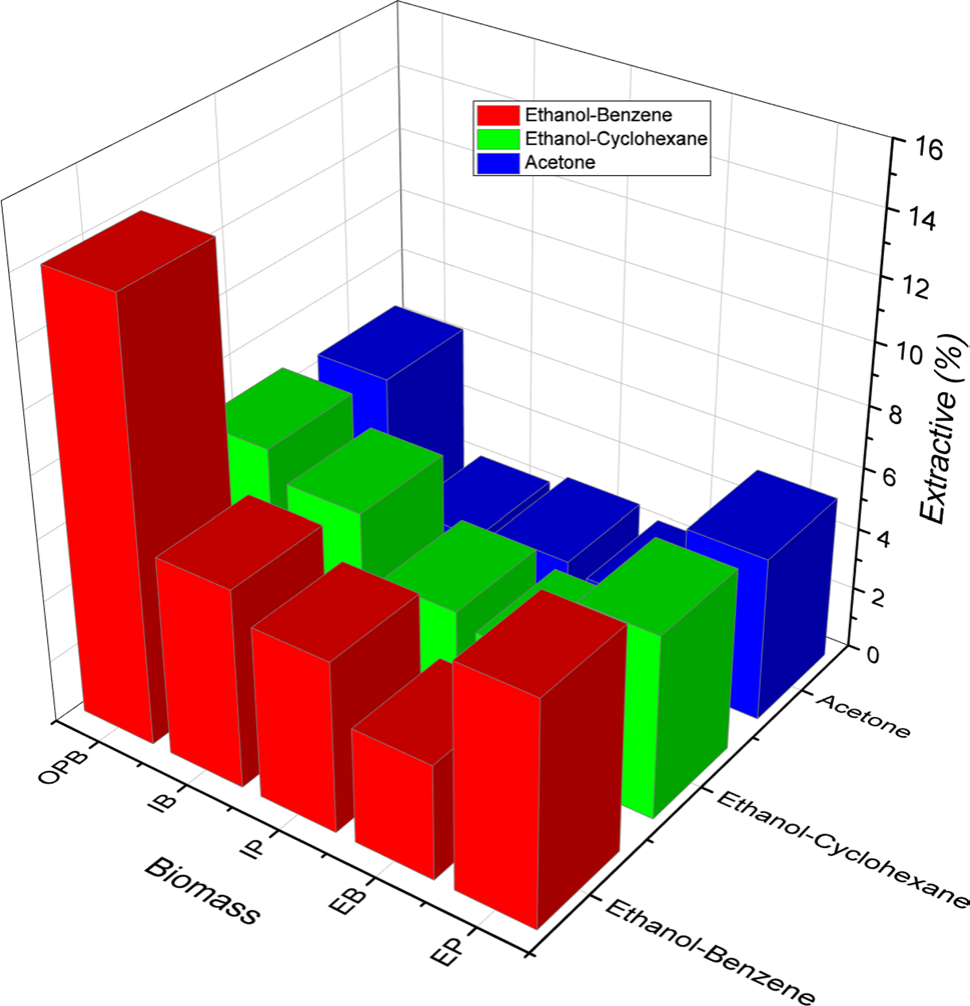
Pre-treatment of biomass with different solvent-mixtures (acetone, ethanol–benzene and ethanol-cyclohexane).

The conventional solvents for extracting these compounds from biomass are ethanol and benzene mixture. Owing to a serious regulatory, environmental, health and safety (EHS) ban presently imposed on benzene, an attempt was made to substitute it with cyclohexane to extract organic matters from the biomass sample. The extraction of the biomass was carried out using different solvents such as acetone, ethanol–benzene mixture and ethanol-cyclohexane. Figure 2 shows extractive yields obtained from using different solvents. Ethanol–benzene had the highest yields ranging from OBP 14%, EP 7%, IB 6.39%, IP 5.6% to EB 3.8%; followed by ethanol-cyclohexane (OBP 6.78%, EP and IB 6%, IP 4%, and EB 3.55%) and least yield was obtained when acetone was used as solvent (OBP 6.6%, EP 5.6%, EB 2.43%, IP 2.8% and IB 2.4%). From the results, percentage amount extracted followed the same trend with respect to the biomasses. It was noted that ethanol-cyclohexane compared closely with the conventional solvent mixture having extracted about 71–94% of what ethanol–benzene extracted in all the biomasses except OBP, and as such could be an environmentally friendly alternative solvent for biomass pre-treatment. However, acetone demonstrated a much lower comparative capacity (38–80%) of what ethanol–benzene extracted, and would therefore not be strongly recommended to replace the conventional solvent.

### Physicochemical properties of wild *Dioscorea bulbifera*

Physicochemical composition properties of *D. bulbifera* biomass was determined in accordance with methods employed in wood chemistry. The results obtained from analyses are summarized in Table 2.

**Table 2:** Physicochemical properties of lignocellulosic biomass.

Biomass	Bulk density	Tapped density	Moisture content	pH
EP	0.19^a^ ± 0.01	0.29^b^ ± 0.00	0.92^a^ ± 0.02	7.20^b^ ± 0.10
EB	0.55^c^ ± 0.15	0.57^c^ ± 0.03	0.92^a^ ± 0.00	7.50^c^ ± 0.60
IP	0.39^ab^ ± 0.37	0.23^a^ ± 0.00	0.92^a^ ± 0.01	6.20^a^ ± 0.26
IB	0.47^ab^ ± 0.01	0.23^a^ ± 0.00	0.92^a^ ± 0.02	7.40^bc^ ± 0.10
OBP	0.41^ab^ ± 0.01	0.52^c^ ± 0.01	0.92^a^ ± 0.02	7.60^c^ ± 0.14

Means ± standard deviation of triplicate determinations. Means within a column with the same superscript letters are not significantly different (p > 0.05) by Duncan’s multiple range test.

*EP* Ekiti peels of *Dioscorea bulbifera,* *EB* Ekiti bulbils of *Dioscorea bulbifera,* *IP* Isarun peels of *Dioscorea bulbifera,* *IB* Isarun bulbils of *Dioscorea bulbifera,* *OPB* Ondo peels and bulbils of *Dioscorea bulbifera.*

#### Moisture content

Moisture content was 0.92% for all the biomass implying they all have equal inherent water retention properties.

#### Bulk and tapped densities

Bulk and tapped densities are a measure of how well powdered samples could be compacted and compressed in a confined space. EB biomass had the highest bulk density of 0.55 and tapped density of 0.57 g/cm^3^ and thus has the highest compatibility and compressibility properties as shown in Table 2. Bulk densities were 0.19 g/cm^3^ for EP, 0.39 g/cm^3^ for IP, 0.47 g/cm^3^ for IB and 0. 41 g/cm^3^ for OBP while tapped density are 0.29 g/cm^3^ for EP, 0.23 g/cm^3^ for IP, 0.23 g/cm^3^ for IB and 0.52 g/cm^3^. The values obtained for tapped density agreed with the stipulated 0.25–0.5 g/cm^3^ of India standard. Generally, bulk and tapped densities are required in biomass if it will be expected to flow and rearrange under compression.

#### pH

Results obtained for pH are arranged in an increasing order of alkalinity: IP < EP < IB < EB < OBP. Expectedly, the highest pH value was obtained in OBP owing to the fact that both bulbils and peels were collectively analysed in the biomass. It was observed that for both samples from Ekiti and Isarun, the peels had lower pH than their bulbils. There are significant differences between the values as shown in Table 2. All the biomasses were alkaline in nature except for IP with pH of 6.2 and this may be a contribution from the environment from which it was collected.

### Characterization of wild *Dioscorea bulbifera* biomass, isolated cellulose and modified cellulose

Fourier transforms infra-red spectroscopy, scanning electron microscopy with energy dispersive X-ray analysis, thermal gravimetric analysis and X-ray diffractometry were employed in the characterization of wild *D. bulbifera* feedstock, its isolated and modified cellulose.

#### Fourier transforms infrared analyses

**Figure 3:**
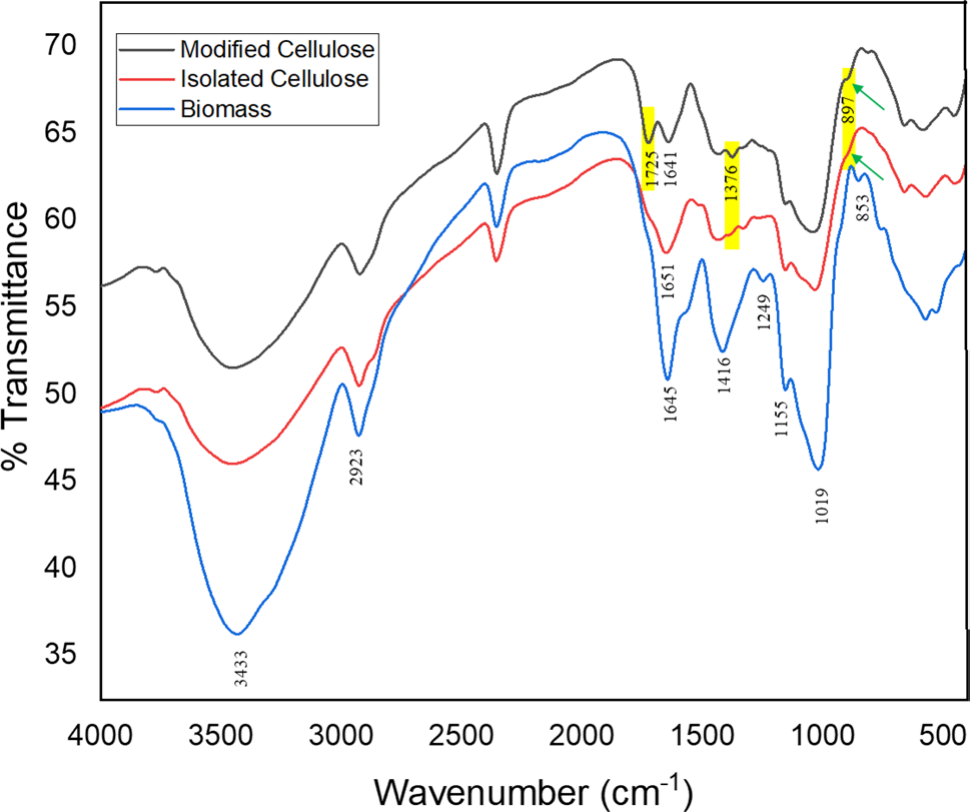
FT-IR spectra for the biomass (*Dioscorea bulbifera*), isolated and modified cellulose.

FT-IR peaks assignments of *D. bulbifera*, its isolated and modified celluloses are shown in Fig. 3. In the biomass spectrum, the prominent peaks seen at 3433 cm^-1^ and 2923.38 cm^-1^ were attributed to the OH stretching of the hydroxyl groups of intra-molecular O(2)H–O(6) and intermolecular O(6)H–O(3) hydrogen bonds vibration and –C–H asymmetric stretching of methylene (–CH_2_) group respectively of the cellulose. The vibration at 1645 cm^-1^ frequency is due to O–H of absorbed water which was accompanied by intensification of the band and is characteristic of conjugation with aromatic ring. The band at 1249 cm^-1^ and 1416.17 cm^-1^ peaks were assigned to the skeletal –C–C-stretching vibration and –C=C-conjugated bonds respectively. The vibration around 1155.68 cm^-1^ is corresponding to artificial strong non-symmetrical bridge –C–O–C– of amorphous cellulose presence in biomass while the peaks at 1019.33 cm^-1^ and 853.47 cm^-1^ were attributed to C–H aromatic in plane bending and O–H out of plane bending on aromatic ring respectively.

In the isolated and modified celluloses spectra, 1651 and 1641 cm^-1^ are attributed to the vibration of water molecules absorbed in cellulose. This band is known as the crystallinity band. The reduction in intensity of such bands indicated that the side chain of lignin was broken down during chemical treatment. The peak at 1376 cm^-1^ is assigned to C-H bending of cellulose. An increase in the intensity of 1376 cm^-1^ and 897 cm^-1^ bands as indicated in spectra is characteristic of transformation to a mercerized cellulose^36^. The new peak at 1725 cm^-1^ is related to un-conjugated asymmetric and symmetric carbonyl stretching of ester C=O. This confirms the occurrence of esterification reaction between cellulose and maleic anhydride during synthesis and this implies that the maleic anhydride was successfully introduced onto the cellulose surface.

#### Scanning electron microscopy (SEM) and energy dispersive X-ray spectroscopy (EDX)

**Figure 4:**
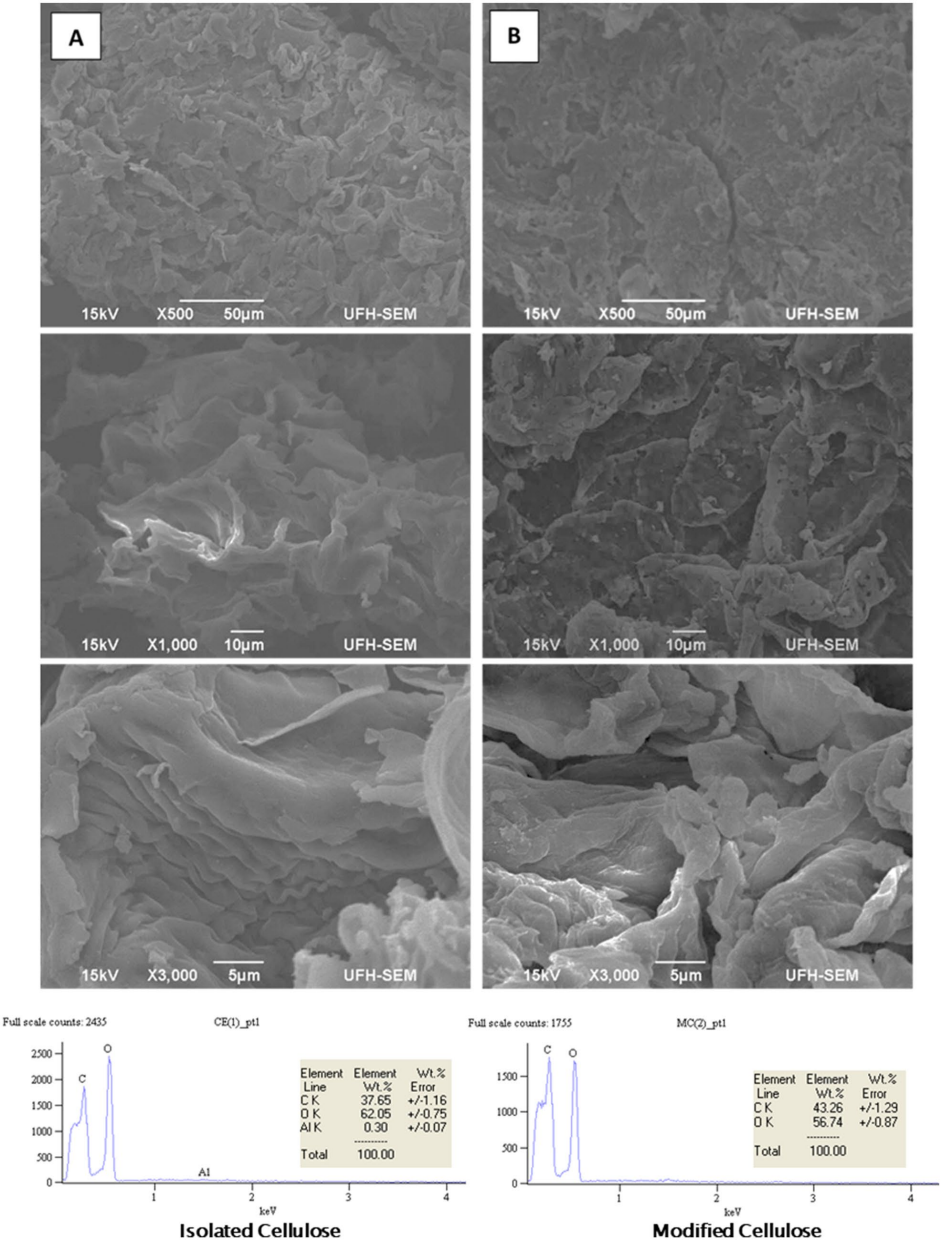
Top: SEM morphology at × 500, 1000 and 3000 of **(a)** isolated cellulose, **(b)** modified cellulose. Bottom: EDX spectra of isolated and modified cellulose.

SEM was used to observe the microstructural morphology of the isolated cellulose and modified cellulose. Figure 4 (top) at magnification (500×) shows the roughness and small pore sizes surface of both isolated cellulose and modified cellulose. At 1000× magnification, the isolated cellulose appeared as a clustered sheet and a little rougher compared to modified cellulose with separated perforated sheet which has irregular pores. At magnification (3000×), overlapped/compacted and ordered chain folding in the isolated cellulose was much more pronounced, while random, wavy chain folding was observed in the modified cellulose. This was as a result of alkaline treatment with the isolated cellulose which opened up the microfibrils of the cellulose.

EDX spectral results (Fig. 4 bottom) revealed that C, O and Al are the main inorganic elements in the celluloses. The presence of Al (0.30 wt%) in the isolated celluloses could not have been from the soil from which the biomass was harvested. Rather, it could be from trace metal impurities in the reagents used in processing the biomass at the pre-treatment stages. Expectedly, it was observed that the concentration of elemental C was higher in modified cellulose (43.26 wt%) when compared with that of isolated cellulose (37.65 wt%) while the elemental O reduced in modified cellulose (56.74 wt%) when compared with its concentration in the isolated cellulose (62.05 wt%). The trend observed could be traced to the fact that upon modification with maleic anhydride, three (3) atoms of O were replacing one (1) atom of O from the hydroxyl group while four (4) atoms of C were being added.

#### XRD analysis results of cellulosic material and modified cellulose

**Figure 5:**
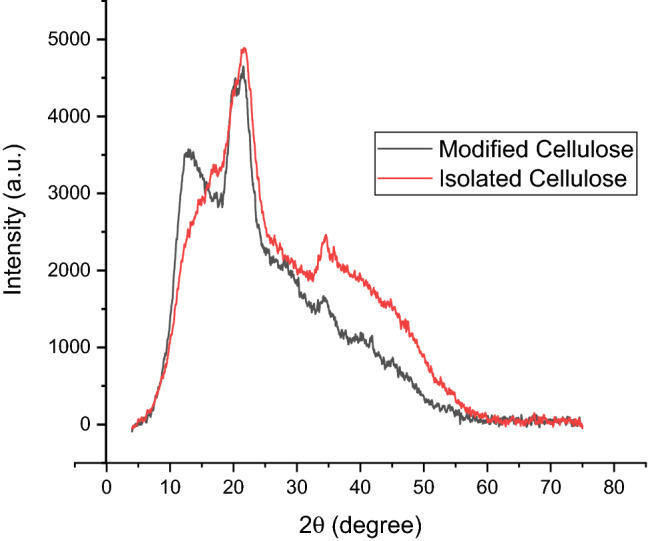
XRD of isolated cellulose (red) and modified cellulose (black) respectively.

The X-ray diffraction pattern of the isolated cellulose shown in Fig. 5 exhibited main reflection peaks relative to cellulose crystal structure at 2Ɵ = 17.2° (110 plane), 21.5° (200 plane) and 34.6° (004 plane) with a crystallinity index (CI) of 98.7%. Modified cellulose showed main reflection peaks at 2Ɵ = 17.3° (110 plane) and 21.5° (200 plane) and 34.4° (004 plane) with CI of 95.5%. The CI was estimated from the integration of the areas of amorphous and crystalline regions in the sample using Origin 2019b. As shown in Fig. 5, the intensities of the peaks at the main reflections reduced in the modified cellulose leading to reduced degree of CI of cellulose after modification. Modification, especially, mercerization reaction, is known to impart more amorphous property to cellulose. This agrees with the SEM results earlier discussed in 3.3.2 and similar results reported by others ^37^. Determination of the crystallite size of both celluloses was attempted using Scherrer’s equation and it showed average crystallite sizes were 1.39 nm and 1.01 nm for modified cellulose and isolated cellulose respectively. This marginal increase in crystal size observed for the modified cellulose resulted from swelling effect imparted upon cellulose microfibrils during mercerization subsequent to esterification with maleic anhydride.

#### Thermal gravimetric analysis of cellulose and modified cellulose

**Figure 6:**
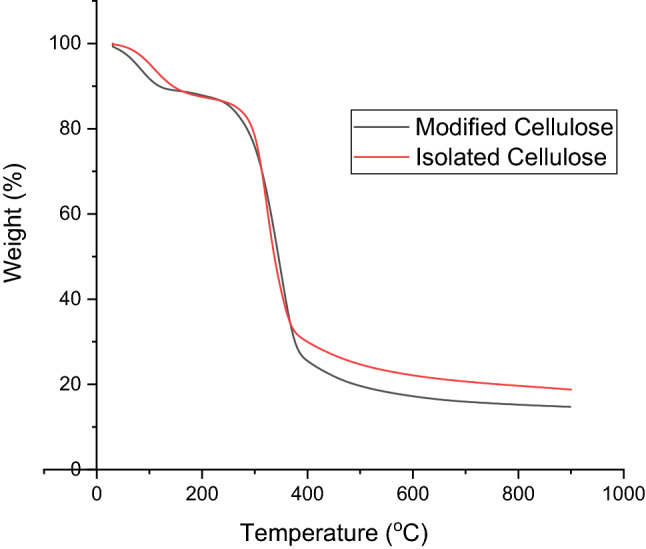
TGA profiles of Isolated cellulose and modified cellulose.

Thermal behaviour of both isolated and modified celluloses is presented in Fig. 6. There were no major differences between the thermal behaviour of isolated and modified cellulose. The initial weight loss for both samples occurs below 100 °C which was attributed to the removal of absorbed water. As shown in Fig. 6 the second weight loss, which started at about 267 °C for isolated cellulose and 237 °C for modified cellulose, signified the initial stage of cellulose thermal degradation. The rapid weight loss, which continued till 380 °C reflects major thermal degradation which is attributed to the thermal cleavage of the glycosidic units and scission of the C–O bonds^38^. Results revealed that the modified cellulose has relatively lower stability compared to the isolated. At about 20% and 70% weight loss, degradation was (284 °C, 377 °C) and (295 °C, 414 °C) respectively for modified and isolated celluloses. This again corroborates the marginal CI reduction observed in the modified cellulose using XRD data.

## Conclusions

This study showed the conversion of poisonous wild *Dioscorea bulbifera* to economic-friendly, potentially-biodegradable and useful resources. First, it was observed that chemical composition of the biomass varies from one location to another. Secondly, ethanol-cyclohexane solvent mixture was performed as a close greener pre-treatment solvent replacement for ethanol–benzene owing to toxicity of benzene. Additionally, chemical analyses of the components of the biomass revealed its suitability as pulp and paper manufacturing materials, though more analyses will be required to substantiate this. More importantly, the modified cellulose (cellulose maleate) prepared from the biomass showed structural and morphological properties that presents applications as adsorbents and industrial materials.
